# Decreased Frequencies of Gamma/Delta T Cells Expressing Th1/Th17 Cytokine, Cytotoxic, and Immune Markers in Latent Tuberculosis-Diabetes/Pre-Diabetes Comorbidity

**DOI:** 10.3389/fcimb.2021.756854

**Published:** 2021-10-26

**Authors:** Gokul Raj Kathamuthu, Nathella Pavan Kumar, Kadar Moideen, Pradeep A. Menon, Subash Babu

**Affiliations:** ^1^National Institutes of Health-NIRT-International Center for Excellence in Research, Chennai, India; ^2^Indian Council of Medical Research-National Institute for Research in Tuberculosis (ICMR-NIRT), Chennai, India; ^3^Laboratory of Parasitic Diseases, National Institute of Allergy and Infectious Diseases, National Institutes of Health, Bethesda, MD, United States

**Keywords:** γδ T cells, Th1/Th17 cytokines, cytotoxic and immune markers, latent tuberculosis, diabetes mellitus, pre-diabetes

## Abstract

Antigen-specific gamma-delta (γδ) T cells are important in exhibiting anti-mycobacterial immunity, but their role in latent tuberculosis (LTB) with diabetes mellitus (DM) or pre-DM (PDM) and non-DM comorbidities have not been studied. Thus, we have studied the baseline, mycobacterial (PPD, WCL), and positive control antigen-stimulated γδ T cells expressing Th1 (IFNγ, TNFα, IL-2) and Th17 (IL-17A, IL-17F, IL-22) cytokine as well as cytotoxic (perforin [PFN], granzyme [GZE B], granulysin [GNLSN]) and immune (GMCSF, PD-1, CD69) markers in LTB (DM, PDM, NDM) comorbidities by flow cytometry. In the unstimulated (UNS) condition, we did not observe any significant difference in the frequencies of γδ T cells expressing Th1 and Th17 cytokine, cytotoxic, and immune markers. In contrast, upon PPD antigen stimulation, the frequencies of γδ T cells expressing Th1 (IFNγ, TNFα) and Th17 (IL-17F, IL-22) cytokine, cytotoxic (PFN, GZE B, GNLSN), and immune (CD69) markers were significantly diminished in LTB DM and/or PDM individuals compared to LTB NDM individuals. Similarly, upon WCL antigen stimulation, the frequencies of γδ T cells expressing Th1 (TNFα) and Th17 (IL-17A, IL-22) cytokine, cytotoxic (PFN), and immune (PD-1, CD69) markers were significantly diminished in LTB DM and/or PDM individuals compared to LTB NDM individuals. Finally, upon P/I stimulation we did not observe any significant difference in the γδ T cell frequencies expressing cytokine, cytotoxic, and immune markers between the study populations. The culture supernatant levels of IFNγ, TNFα, and IL-17A cytokines were significantly increased in LTB DM and PDM after stimulation with Mtb antigens compared to LTB NDM individuals. Therefore, diminished γδ T cells expressing cytokine, cytotoxic, and other immune markers and elevated levels of cytokines in the supernatants is a characteristic feature of LTB PDM/DM co-morbidities.

## Introduction

Tuberculosis, caused by *Mycobacterium tuberculosis* (Mtb), affects around 10 million people and causes 1.4 million deaths worldwide in the year 2019 (World Health Organization, 2020). Latent tuberculosis (LTB) is defined as an asymptomatic clinical state without any infection or not transmissible, whereas active TB is defined by the presence of clinical symptoms and affects different parts of the body (Barry et al., 2009). Globally, one-quarter of the world’s population is infected with LTB (Dheda et al., 2016). In addition to this, type 2 diabetes mellitus (DM) comorbidity enhances the risk of TB development by three times (Restrepo, 2016). Hence, a higher risk of active TB and reactivation of LTB infection has long been well-known to be linked with the risk of DM (Siddiqui et al., 2016). In India, DM prevalence is calculated to reach 123.5 million by 2040 (International Diabetes Federation, 2015). Similarly, 5%–10% of the pre-diabetes mellitus (PDM) or intermediate hyperglycemic individuals become diabetic every year depending on the inhabitants and geographical location (Forouhi et al., 2007; Abdul-Ghani and DeFronzo, 2009). PDM prevalence is shown to be as high as 25% of individuals among active TB (Viswanathan et al., 2012).

Type 1 (IFNγ and TNFα), type 17 (IL-17), IL-1, and IL-12 family cytokines are thought to be crucial for host protection against pulmonary tuberculosis (PTB) (O’Garra et al., 2013; Mayer-Barber and Sher, 2015). TB DM comorbidity is associated with enhanced plasma and antigen-specific levels of pro-inflammatory cytokines (Restrepo et al., 2008; Kumar et al., 2013). LTB DM is associated with decreased systemic and Mtb antigen-specific levels of type 1 and type 17 cytokines (Kumar et al., 2014). The previous study has also shown that LTB PDM was characterized by decreased antigen-specific frequencies of T helper (Th)1/T cytotoxic (Tc)1 and Th17/Tc17 cells (Kumar et al., 2017). Likewise, non-conventional T cells play a crucial function in protecting against TB disease, especially in early infection stages (De Libero and Mori, 2014; Huang, 2016; Meermeier et al., 2018). Among them, γδ T cells are innate-like T lymphocytes that comprise a small segment (1%–5%) of the circulating T lymphocytes and play a vital role in triggering host cell-mediated immune responses (Hiromatsu et al., 1992; Ribeiro et al., 2015). Antigen-specific γδ T cells constitute a primary innate immune defense that could play an important function in anti-mycobacteria-mediated immunity. They are also able to recognize Mtb antigens by responding to Bacille Calmette–Guerin (BCG) vaccination, inhibiting mycobacterial growth, and providing protective immunity upon adoptive transfer (Balbi et al., 1993; Zhang et al., 2006; Brandes et al., 2009; Price and Hope, 2009). Recently, upon moderate-dose TB challenge, the expansion of Vγ2Vδ2 T cells was observed and correlated with decreased TB infection and disease pathology in non-human primates (NHPs) vaccinated with attenuated HMBPP-producing *Listeria monocytogenes* (Shen et al., 2019).

Recent data suggest that reduced frequencies in γδ T cells are associated with reduced survival rate and increased lung injury upon H5N1 respiratory infection (Dong et al., 2018). It was also previously described that influenza-affected individuals require γδ T cells for effective immunity and maintenance of lung homeostasis (Xi-zhi et al., 2018; Sabbaghi et al., 2020). γδ T cell exhaustion results in impaired host defense to lung infections by different pathogens like *Klebsiella pneumonia*, *Streptococcus pneumoniae*, and *Staphylococcus aureus* (Moore et al., 2000; Kirby et al., 2007; Cho et al., 2010). Although γδ T cells are considered to be the primary orchestrator of immune responses in TB disease and DM known to increase the clinical disease severity and affect treatment response, the mechanism of DM or PDM comorbidities in altering the disease severity and γδ T cell-induced correlates of protective immunological response in LTB disease is not known. Especially, the roles of γδ T cell immune responses in LTB DM, pre-DM (PDM), and non-DM (NDM) comorbidity are not characterized.

Hence, we measured the frequencies of γδ T cells expressing Th1 (IFNγ, TNFα, IL-2) and Th17 (IL-17A, IL-17F, IL-22) cytokines, cytotoxic markers [perforin (PFN), granulysin (GNLSN), granzyme (GZE) B], and other immune markers (GMCSF, PD-1, CD69) using immune phenotyping by flow cytometry. In addition, we measured the Mtb antigen-stimulated supernatants of IFNγ, TNFα, and IL-17A cytokines between the LTB comorbidities. We reveal that γδ T cells expressing Th1, Th17, cytotoxic, and immune markers were significantly diminished in LTB DM and/or PDM compared to LTB NDM-coinfected individuals. The antigen-stimulated supernatant levels of IFNγ, TNFα, and IL-17A cytokines were significantly increased between the study individuals. Overall, our data suggest that diminished frequencies of γδ T cells expressing cytokines and cytotoxic markers and elevated cytokines in the supernatants are an important characteristic feature in LTB DM and PDM comorbidities.

## Materials and Methods

### Study Group

The study was sanctioned by the National Institute of Research in Tuberculosis (NIRT) Internal Ethics Committee (NIRTIEC2011013), and informed written consent was obtained from all study individuals. A group of 60 patients were recruited with LTB disease and separated into three different arms (20 individuals in each arm) based on their diabetes status as follows: diabetes mellitus (DM), pre-DM (PDM), and non-DM (NDM) (**Table 1**). LTB positivity was defined by both tuberculin skin test (TST) and QuantiFERON-TB GOLD in tube ELISA. These individuals did not have any active tuberculosis pulmonary symptoms and possessed normal chest radiographs. The TST positive was expressed as an induration at the site of tuberculin inoculation of at least 12 mm in diameter to minimize false positivity occurrence owing to contact with environmental mycobacteria. Type 2 DM (HbA1c levels > 6.5%, random blood glucose > 200 mg/dl), PDM (HbA1c levels >5.7% to <6.4%, random blood glucose 140–199 mg/dl), and NDM (HbA1c levels <5.7% random blood glucose <140 mg/dl) were detected on the basis of glycated hemoglobin (HbA1c) levels and random blood glucose according to the criteria given by the American Diabetes Association. The study individuals were BCG vaccinated, HIV negative, and not under the usage of any steroids and did not have any signs or symptoms of related lung or systemic disease.

**Table 1:** Demographics of the study population.

Study demographics	LTB NDM	LTB PDM	LTB DM
**Number of subjects recruited (n)**	20	20	20
**Gender (M/F)**	10/10	11/9	11/9
**Median age in years (range)**	39.6 (24–62)	45.9 (25–62)	47.1 (25–60)
**Glycated hemoglobin level, %**	<5.48 (5.0–5.9)	>6.1 (5.9–6.3)	8.43 (6.50–11.96)
**QuantiFERON-TB gold assay**	Positive	Positive	Positive

### Isolation of Peripheral Blood Mononuclear Cell Isolation and Thawing

Peripheral blood mononuclear cells (PBMCs) were isolated from the collected peripheral blood samples using Ficoll-Paque (GE Life Sciences) density gradient centrifugation for 30 min at 400 × g at 4°C, and the cells were stored at -80°C using fetal bovine serum (FBS) and dimethyl sulfoxide (DMSO). The PBMC cells were thawed in 37°C in a water bath using a thawing [Roswell Park Memorial Institute 1640 (nine parts), RPMI-FBS (one part)] medium.

### Mycobacterial Antigens and Stimulation

The antigens used are purified protein derivative (PPD; Statens Serum Institut, 10 μg/ml), whole-cell lysate (WCL 1 μg/ml), and phorbol 12-myristate 13-acetate (P)/ionomycin (P/I; Calbiochem, 12.5 and 125 ng/ml). The thawed PBMC cells were either unstimulated or stimulated with mycobacterial (Mtb) antigens and incubated for 18 h at 37°C in 5% CO_2_. FastImmune™ brefeldin A (10 μg/ml) was added after 2 h of post-incubation. Once incubation was completed, the cells were transferred into sterile falcon tubes, washed with PBS, and dissolved using a permeabilization buffer, and cell staining was performed. The cultured PBMC supernatants were collected and used for ELISA.

### PBMC Staining and Flow Cytometry

The thawed cells were washed with PBS first and PBS/1% BSA later, then stained with surface [anti-CD3-AmCyan clone-SK7 (also known as Leu-4) (ASR), catalog no.: 339197, BD Biosciences], anti-CD69 (PB, anti-human, clone-FN50, catalog no: 310920, BioLegend), and anti-CD279 (PD-1) (PB, anti-human, clone-EH12.2H7, catalog no: 329916, BioLegend)] antibodies for 30–60 min at 4°C in the dark. Next, the cells were washed and permeabilized with 2 ml of 1× BD Perm/Wash buffer (BD Biosciences) and stained with intracellular cytokines for an additional 2 h at 4°C before washing (1× permeabilization buffer) and acquisition. The intracellular antibodies used in the study are as follows: γδ TCR (PE-Cyanine7, anti-human, clone-B1, BioLegend, catalog no: 331222), αβ TCR (PerCp/Cyanine5.5, anti-human, clone-IP26, BioLegend, catalog no: 306724), TNFα (FITC, mouse anti-human, clone-6401.1111 [RUO-(GMP)] catalog no: 340511, BD Pharmingen), IFNγ (PE, mouse anti-human, clone-B27 catalog no: 562016, BD Pharmingen), IL-2 (APC, human monoclonal antibody, clone-MQ1-17H12, catalog no: 17-7029-41, eBioscience™), IL-17A (FITC, anti-human, clone-CZ8-23G1, catalog no: 130-120-550, Miltenyi Biotec), IL-22 [PE, monoclonal antibody, clone-22URPI, catalog no: 12-7229-42, Invitrogen (eBioscience)], IL-17F (Alexa Fluor 647, mouse anti-human, clone-O33-782, catalog no: 561333, BD Biosciences), perforin (FITC, anti-human, clone-delta G9, catalog no: 130-096-668, Miltenyi Biotec), granzyme B [Alexa Fluor 647, mouse anti-human, clone-GB11, catalog no: BDB560212, Invitrogen (eBioscience)], granulysin [PE, anti-human, clone-DH2, catalog no: 12-8828-42, Invitrogen (eBioscience)], and granulocyte macrophage-colony-stimulating factor [GM-CSF] [pacific blue (PB), anti-human, clone-BVD2-21C11, catalog no: 502314, BioLegend]. For multicolor flow cytometry analysis, the stained PBMCs (CD3^+^γδ^+^ T cells) were acquired using an eight-color FACSCanto II flow cytometer with FACSDiva software v.6 (Becton Dickinson and Company, Cockeysville, MD). The lymphocyte gating was set using forward *vs.* side scatter, and 50,000 gated lymphocyte events were acquired. The gating strategy for γδ T cells expressing Th1 and Th17 cytokines and cytotoxic and immune markers was determined by FMO and exemplified as frequencies (unstimulated) and net frequencies (antigen stimulation).

### ELISA

We measured the unstimulated, Mtb antigen (PPD, WCL), and P/I-stimulated levels of IFNγ, TNFα, and IL-17A cytokines using ELISA (DuoSet Kits R&D Systems). The detection limit of IFNγ is 9.375–600 pg/ml, that of TNFα is 15.625–1,000 pg/ml, and that of IL-17A is 15.625–1,000 pg/ml.

### Data Analysis

The cytokine and cytotoxic marker frequencies of γδ T cell subsets were analyzed using FlowJo 3 (version 10, [Treestar, Ashland, OR]) software. The statistical analysis was carried out using GraphPad Prism (version 9) (GraphPad Software, Inc., San Diego, CA). Geometric means (GM) were used to measure the central tendency, and intergroup comparisons were analyzed by the non-parametric Kruskal–Wallis test. The Mtb antigen and P/I stimulation cytokine levels were represented as net cytokine values. For heatmap analysis, the frequencies of cytokines and cytotoxic and immune markers were normalized and the data are shown using GraphPad Prism with p < 0.05 (ANOVA) considered as statistically significant. The smallest value in each data set is defined as 0%, and the highest value in the dataset is defined as 100%.

## Results

### LTB DM/PDM Comorbidities Are Associated With Reduced γδ T Cells Expressing Th1 Cytokines

**Figure 1:**
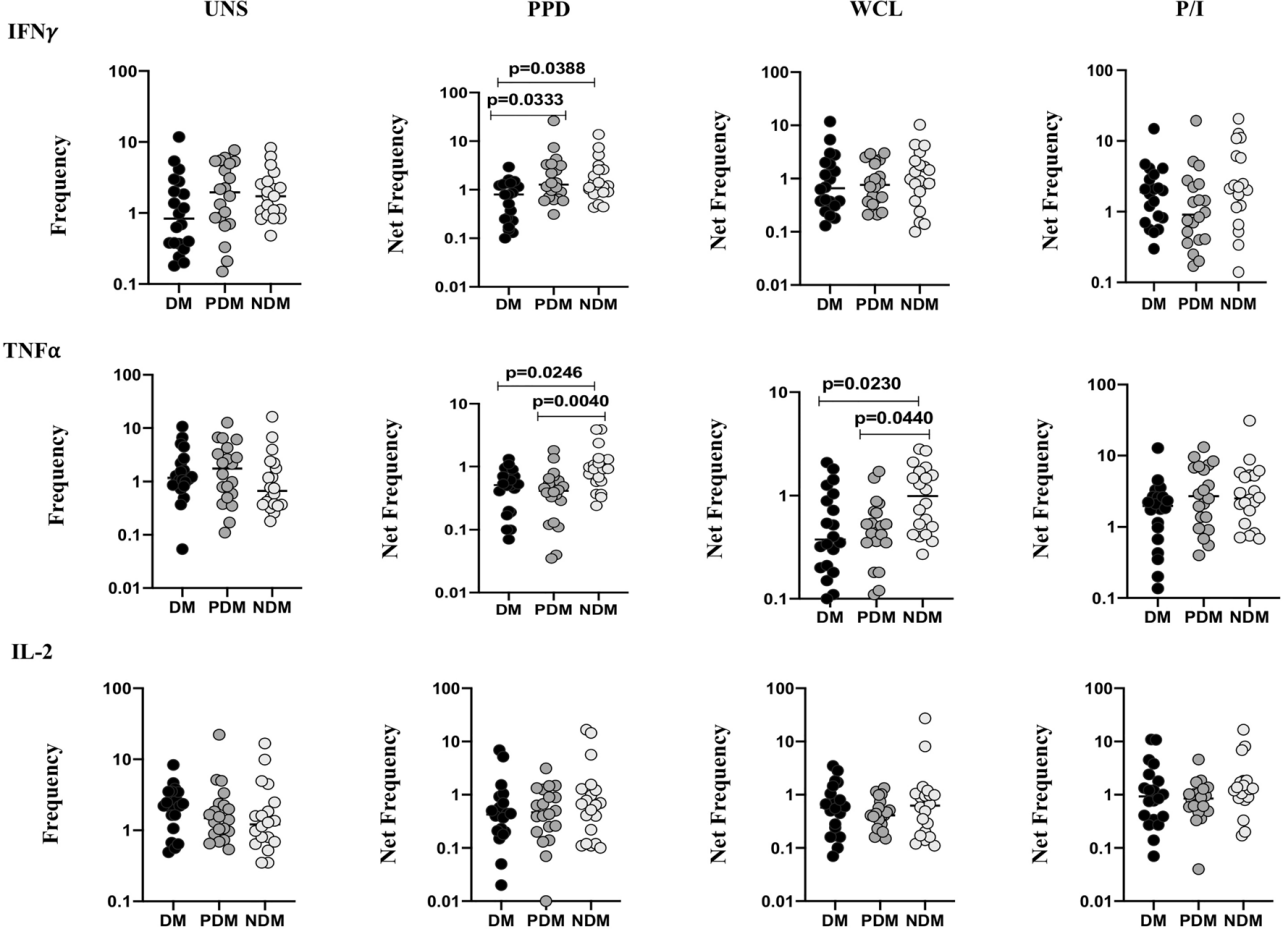
Decreased frequencies of γδ T cells expressing Th1 cytokines in LTB comorbidities. Peripheral blood mononuclear cells (PBMCs) were either untreated or treated with Mtb or positive control antigens for 18 h. The absolute (unstimulated, UNS) and antigen-stimulated (PPD, WCL, P/I) net frequencies of Th1 (IFNγ, IL-2, TNFα) cytokines were shown in LTB DM (n = 20), LTB PDM (n = 20), and LTB NDM (n = 20) groups. Geometric mean values were represented using bars, and every circle denotes a single individual. Kruskal–Wallis test with multiple Dunn’s comparison was used to determine the p values.

The gating strategy and representative plots of γδ T cells expressing Th1 (IFNγ, IL-2, TNFα) and Th17 (IL-17A, IL-17F, IL-22) cytokines as well as cytotoxic [perforin (PFN), granzyme (GZE) B, granulysin (GNLSN)] and immune (GMCSF, PD-1, CD69) markers are shown (**Supplementary Figure 1** and **Supplementary Figures 2A–C**). We determined the baseline (unstimulated, UNS) and Mtb antigen (PPD, WCL) and positive (P/I) control-stimulated frequencies of γδ T cells expressing Th1 cytokines in LTB (NDM, PDM, and DM) comorbid individuals using multicolor flow cytometry (**Figure 1**). In UNS stimulation, the frequencies of γδ T expressing Th1 cytokines were not significantly different in LTB coinfected individuals with NDM, PDM, and DM comorbidities. In contrast, the frequencies of γδ T cells expressing Th1 cytokines were significantly reduced in PPD [IFNγ (geometric mean (GM) of DM is 0.568 *versus* (*vs.*) GM of PDM is 1.590 *vs.* GM of NDM is 1.510), TNFα (GM of DM is 0.402 *vs.* GM of PDM is 0.337 *vs.* GM of NDM is 0.906)] and WCL [TNFα (GM of DM is 0.439 *vs.* GM of PDM is 0.449 *vs.* GM of NDM is 0.913)] antigen stimulation in LTB DM and PDM individuals compared to LTB NDM individuals. Finally, upon P/I stimulation, the frequencies of γδ T cells expressing Th1 cytokines were not significantly different among LTB DM, PDM, and NDM individuals (**Figure 1**). Therefore, LTB PDM and DM comorbid individuals are associated with reduced Th1 expressing cytokine frequencies.

### LTB DM/PDM Comorbidities Are Associated With Reduced γδ T Cells Expressing Th17 Cytokines

**Figure 2:**
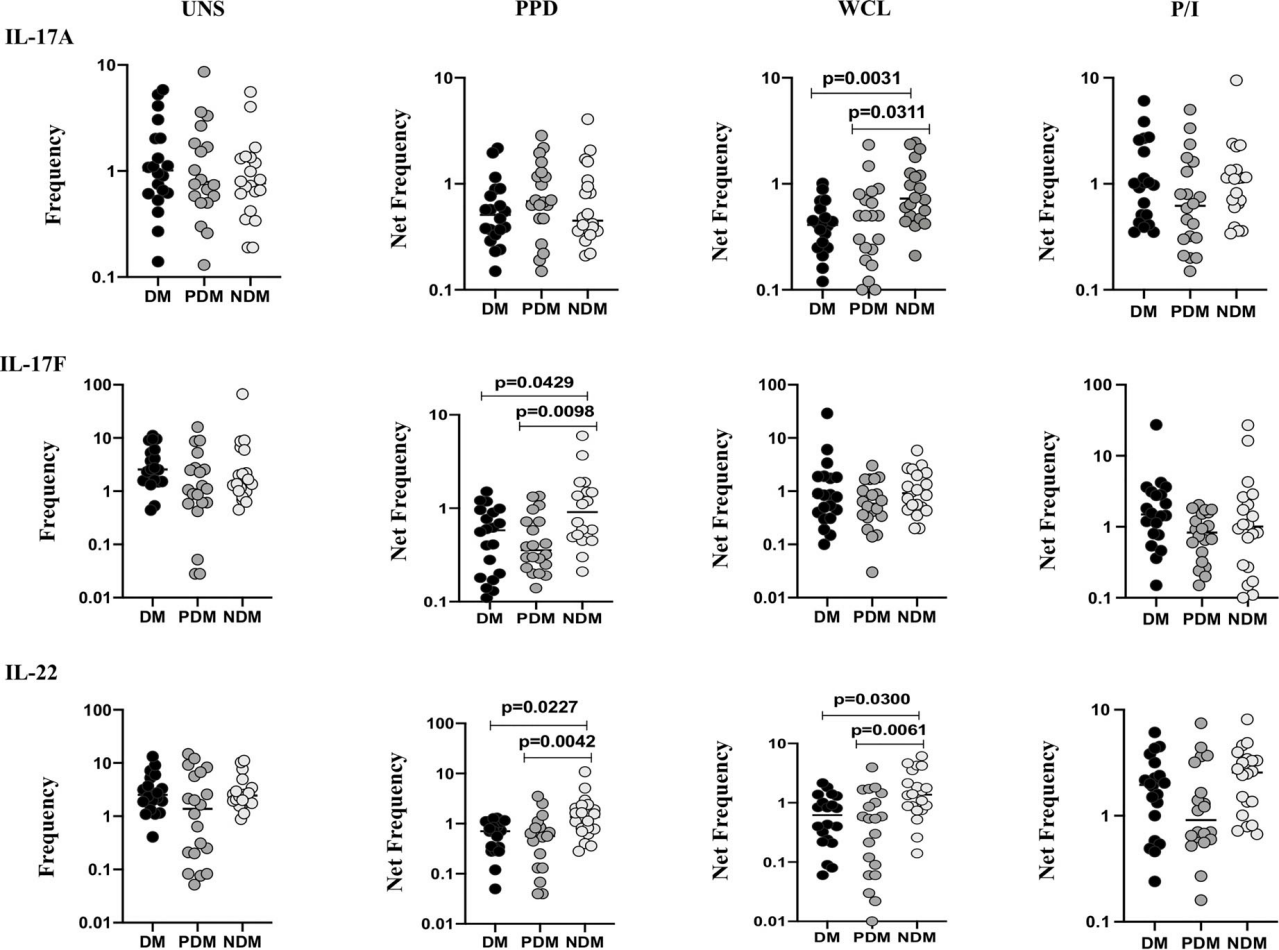
Decreased frequencies of γδ T cells expressing Th17 cytokines in LTB comorbidities. PBMCs were either untreated or treated with Mtb or positive control antigens for 18 h. The absolute (unstimulated, UNS) and antigen-stimulated (PPD, WCL, P/I) net frequencies of Th17 (IL-17A, IL-17F, IL-22) cytokines were shown in LTB DM (n = 20), LTB PDM (n = 20), and LTB NDM (n = 20) groups. Geometric mean values were represented using bars, and every circle denotes a single individual. Kruskal–Wallis test with multiple Dunn’s comparison was used to determine the p values.

We determined the baseline (UNS) and mycobacterial antigen (PPD, WCL) and positive (P/I) control antigen-stimulated frequencies of γδ T cells expressing Th17 (IL-17A, IL-17F, IL-22) cytokines in LTB (NDM, PDM, and DM) comorbid individuals using multicolor flow cytometry (**Figure 2**). In UNS stimulation, the frequencies of γδ T cells expressing Th17 cytokines did not significantly differ between LTB individuals with NDM, PDM, and DM comorbidities. In contrast, the frequencies of γδ T cells expressing Th17 cytokines were significantly reduced in PPD [IL-17F (GM of DM is 0.449 *vs.* GM of PDM is 0.410 *vs.* GM of NDM is 1.022), IL-22 (GM of DM is 0.342 *vs.* GM of PDM is 0.162 *vs.* GM of NDM is 0.848)] and WCL [IL-17A (GM of DM is 0.356 *vs.* GM of PDM is 0.402 *vs.* GM of NDM is 0.807), IL-22 (GM of DM is 0.486 *vs.* GM of PDM is 0.302 *vs.* GM of NDM is 1.329)] antigen stimulation in LTB PDM and DM individuals compared to LTB NDM individuals. Finally, upon P/I stimulation, the frequencies of γδ T cells expressing Th17 cytokines did not exhibit a significant difference between the study population (**Figure 2**). Thus, γδ T cells expressing Th17 expressing cytokine frequencies were significantly diminished in LTB PDM and DM comorbid individuals.

### LTB DM/PDM Comorbidities Are Associated With Reduced γδ T Cell Expressing Cytotoxic Markers

**Figure 3:**
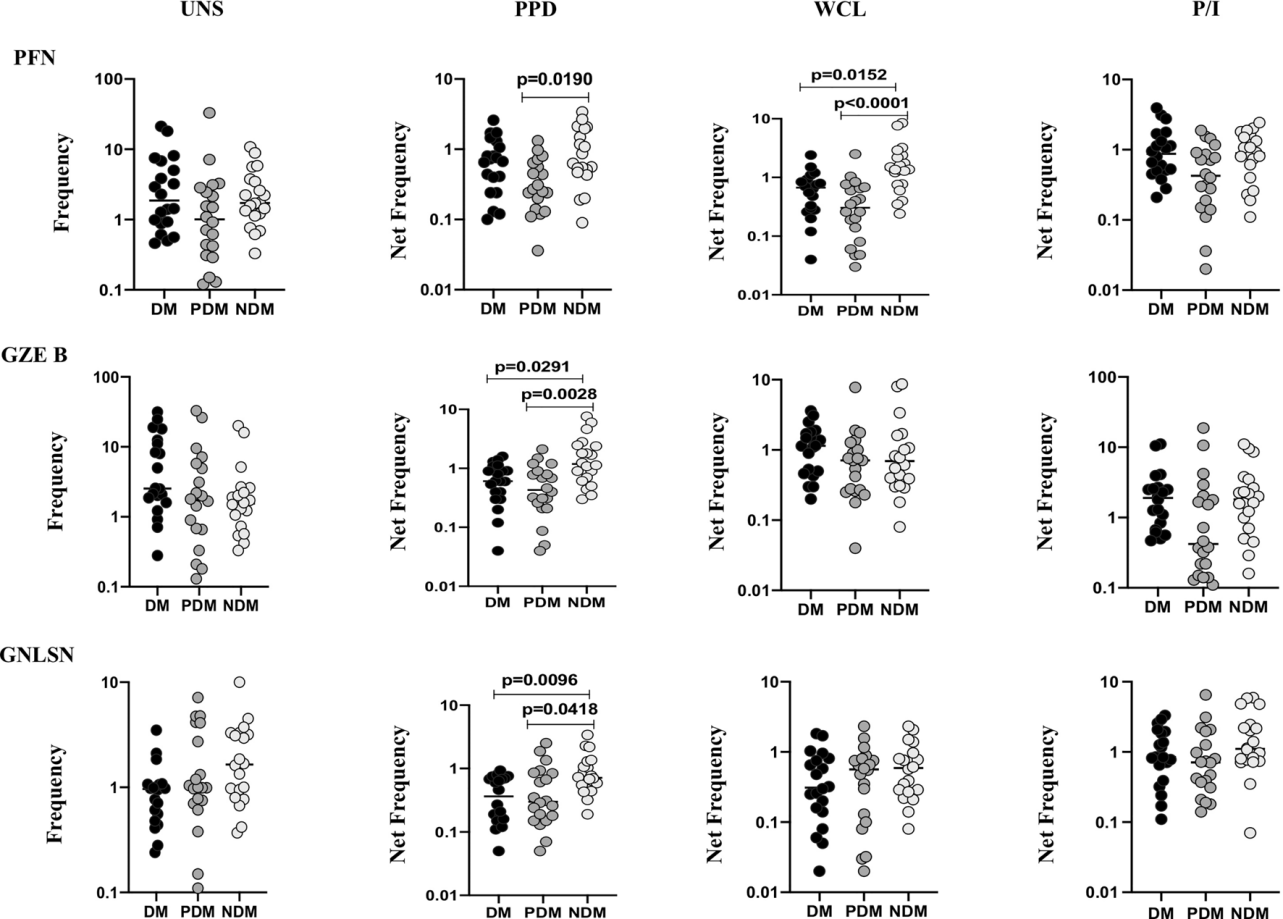
Decreased frequencies of γδ T cells expressing cytotoxic markers in LTB comorbidities. PBMCs were either untreated or treated with Mtb or positive control antigens for 18 h. The absolute (unstimulated, UNS) and antigen-stimulated (PPD, WCL, P/I) net frequencies of cytotoxic (PFN, GZEB, GNLYSN) markers were shown in LTB DM (n = 20), LTB PDM (n = 20), and LTB NDM (n = 20) groups. Geometric mean values were represented using bars, and every circle denotes a single individual. Kruskal–Wallis test with multiple Dunn’s comparison were used to determine the p values.

To determine the γδ T cells expressing cytotoxic markers in LTB individuals with NDM, PDM, and DM comorbidities, we used multicolor flow cytometry to delineate the baseline (UNS) and Mtb antigen-specific (PPD, WCL) stimulated frequencies expressing cytotoxic (perforin (PFN), granzyme B (GZE B), and granulysin (GNLSN) markers) (**Figure 3**). In UNS condition, the frequencies of γδ T cells expressing cytotoxic markers were not significantly altered in LTB PDM and LTB DM individuals compared to LTB NDM individuals. In contrast, net frequencies of γδ T cells expressing cytotoxic markers were significantly reduced upon PPD [PFN (GM of DM is 0.574 *vs.* GM of PDM is 0.295 *vs.* GM of NDM is 0.739), GZE B (GM of DM is 0.533 *vs.* GM of PDM is 0.403 *vs.* GM of NDM is 1.313], GNLSN [GM of DM is 0.316 *vs.* GM of PDM is 0.358 *vs.* GM of NDM is 0.793)] and WCL [PFN (GM of DM is 0.507 *vs.* GM of PDM is 0.265 *vs.* GM of NDM is 1.377)] antigen stimulation in LTB DM and LTB PDM individuals compared to LTB NDM individuals. Finally, upon P/I stimulation, frequencies of γδ T cells expressing cytotoxic markers were not altered in LTB DM and LTB PDM when compared to LTB NDM individuals (**Figure 3**). Therefore, γδ T cells expressing cytotoxic marker frequencies were significantly diminished in LTB DM and PDM individuals.

### LTB DM/PDM Comorbidities Are Associated With Reduced γδ T Cell Expressing Immune Markers

**Figure 4:**
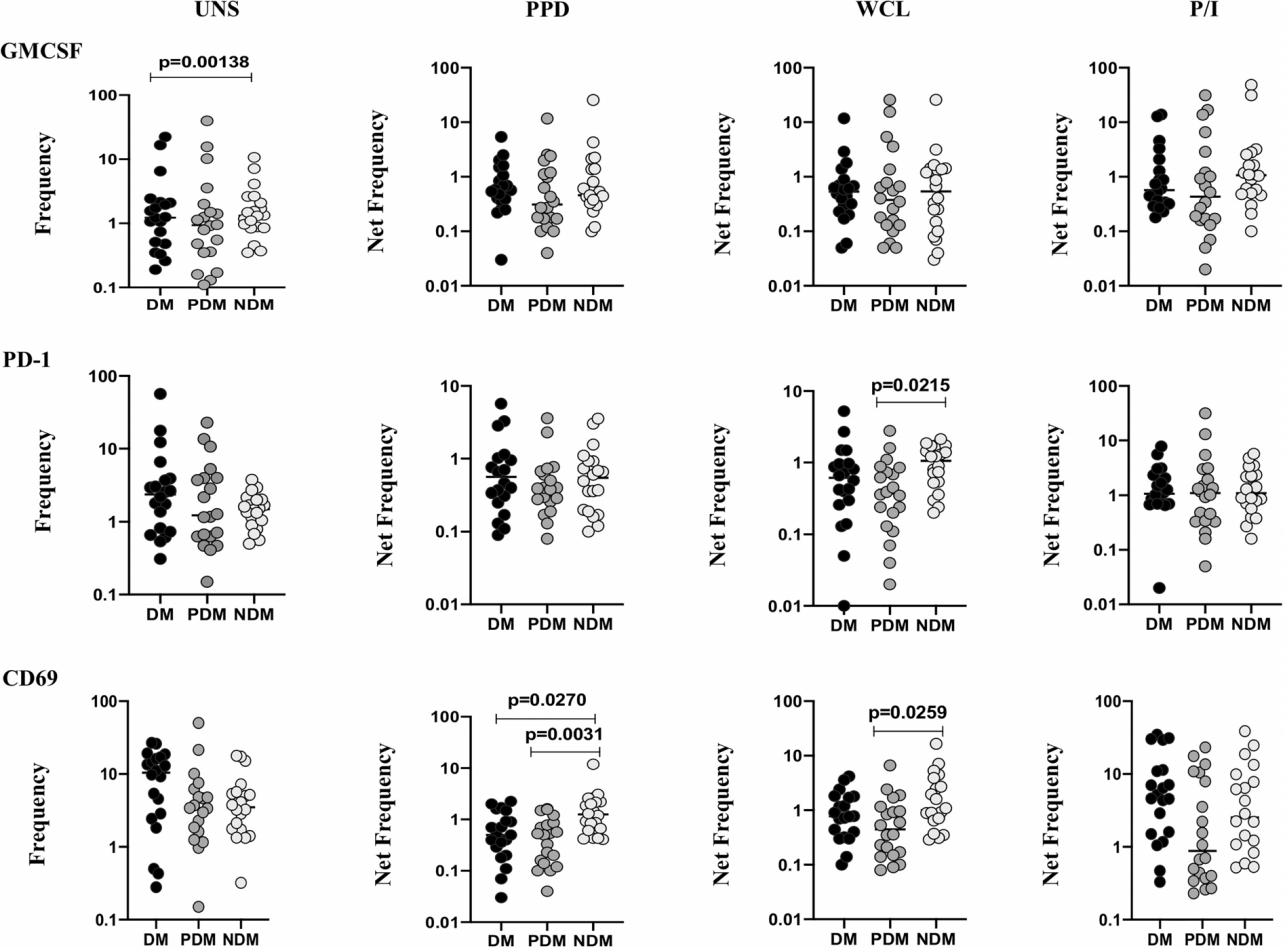
Decreased frequencies of γδ T cells expressing immune markers in LTB comorbidities. PBMCs were either untreated or treated with Mtb or positive control antigens for 18 h. The absolute (unstimulated, UNS) and antigen-stimulated (PPD, WCL, P/I) net frequencies of immune (GMCSF, PD-1, CD69) markers were shown in LTB DM (n = 20), LTB PDM (n = 20), and LTB NDM (n = 20) groups. Geometric mean values were represented using bars, and every circle denotes a single individual. Kruskal–Wallis test with multiple Dunn’s comparison was used to determine the p values.

To determine the γδ T cells expressing immune markers in LTB individuals with NDM, PDM, and DM comorbidities, we used multicolor flow cytometry to delineate the UNS and Mtb antigen-specific (PPD, WCL) stimulated frequencies expressing immune (GMCSF, PD-1, CD69) markers (**Figure 4**). In UNS conditions, the frequencies of γδ T cells expressing immune markers were not significantly altered [except GMCSF (GM of DM is 1.317 *vs.* GM of NDM is 1.437), DM *vs.* NDM] in LTB PDM and LTB DM individuals compared to LTB NDM individuals. In contrast, net frequencies of γδ T cells expressing immune markers were significantly reduced upon PPD [CD69 (GM of DM is 0.468 *vs.* GM of PDM is 0.374 *vs.* GM of NDM is 1.253)] and WCL [PD-1 (GM of DM is 0.469 *vs.* GM of PDM is 0.329 *vs.* GM of NDM is 0.855), CD69 (GM of DM is 0.775 *vs.* GM of PDM is 0.464 *vs.* GM of NDM is 1.393)] antigen stimulation in LTB DM and LTB PDM individuals compared to LTB NDM individuals. Finally, upon P/I stimulation, frequencies of γδ T cells expressing immune markers were not altered in LTB DM and LTB PDM when compared to LTB NDM individuals (**Figure 4**). Therefore, γδ T cells expressing immune marker frequencies were significantly diminished in LTB DM and PDM individuals.

### Elevated PBMC Culture Supernatant Levels of Cytokines in LTB Comorbidities

**Figure 5:**
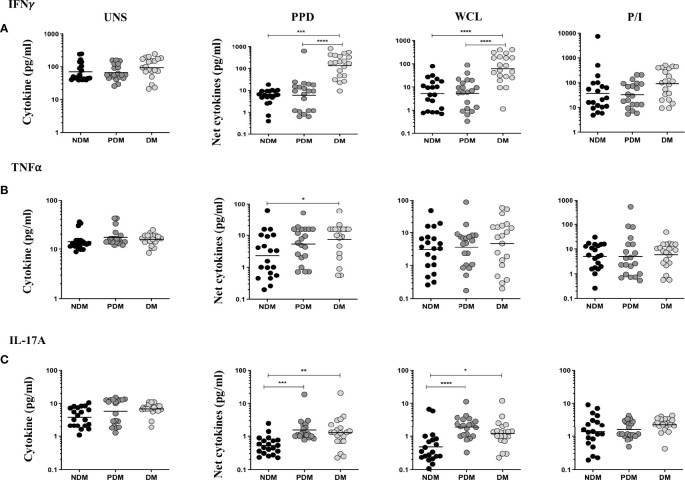
LTB (DM and PDM) coinfection is associated with increased antigen-specific supernatant levels of **(A)** IFNγ, **(B)** TNFα, and **(C)** IL-17A cytokines. PBMCs were cultured with no antigen (UNS), stimulated with Mtb (PPD, WCL), and positive (P/I) control antigens for 18 h in LTB NDM (n = 20), LTB PDM (n = 20), and LTB DM (n = 20) individuals. We measured the net cytokine levels by subtracting the antigen-induced cytokine or cytotoxic and immune markers from their baseline levels for each individual. Geometric mean values were represented using bars, and every circle denotes a single individual. p values were analyzed by Kruskal–Wallis test.

The UNS, Mtb (PPD, WCL) antigen, and P/I-stimulated supernatant levels of IFNγ, TNFα, and IL-17A cytokines in LTB (NDM, PDM, DM) comorbidities are shown (**Figure 5**). As shown in **Figures 5A–C**, the UNS levels of IFNγ, TNFα, and IL-17A cytokines did not significantly differ between the LTB coinfected (NDM, PDM, DM) individuals. However, after stimulation with Mtb antigens, IFNγ [GM of NDM is 6.723 *vs.* GM of PDM is 6.011 *vs.* GM of DM is 95.29 pg/ml in PPD and GM of NDM is 5.243 *vs.* GM of PDM is 5.293 *vs.* GM of DM is 48.99 pg/ml in WCL] supernatant levels were significantly increased in LTB DM and LTB PDM compared to LTB NDM individuals (**Figure 5A**). Similarly, upon stimulation with Mtb antigens, TNFα [GM of NDM is 2.304 *vs.* GM of DM is 7.403 pg/ml in PPD] supernatant levels were significantly increased in LTB DM compared to LTB NDM individuals (**Figure 5B**). Finally, IL-17A [GM of NDM is 0.52 *vs.* GM of PDM is 1.521 *vs.* GM of DM is 1.274 pg/ml in PPD and GM of NDM is 0.475 *vs.* GM of PDM is 1.863 *vs.* GM of DM is 1.195 pg/ml in WCL] supernatant levels were significantly increased in LTB PDM and LTB DM compared to LTB NDM individuals (**Figure 5C**). We did not observe any significant difference upon P/I stimulation between the study individuals (**Figures 5A** **–C**). Hence, LTB-coinfected individuals were associated with increased supernatant levels of IFNγ, TNFα, and IL-17A cytokines.

### Heatmap Analysis of γδ T Cells Expressing Cytokines and Cytotoxic and Immune Markers in LTB Comorbidities

**Figure 6:**
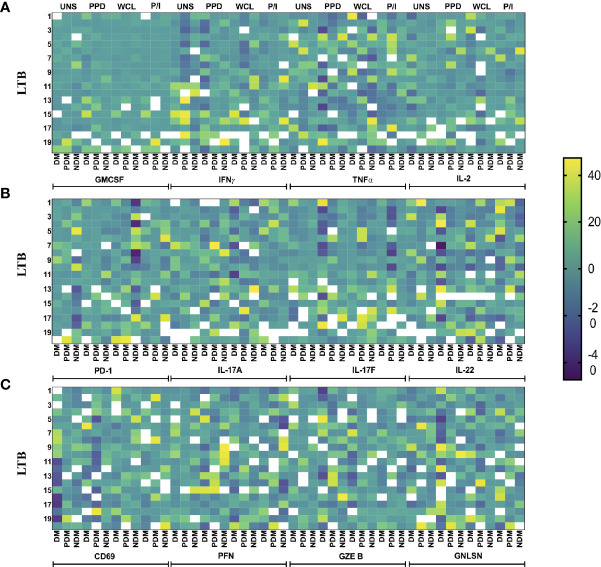
Heatmap analysis of γδ T cells. The cytokine and cytotoxic and immune marker frequencies were normalized across all LTB groups, and the expression data are shown. Each column indicates a single person. We show the heatmap expression of **(A)** GMCSF and Th1 cytokines, **(B)** PD-1 and Th17 cytokines, and **(C)** PD-1 and cytotoxic markers in LTB patients coinfected with DM (n = 20), PDM (n = 20), and NDM (n = 20) individuals upon UNS, Mtb (PPD, WCL), and positive antigen (P/I) control stimulation with continuous color shading.

We performed the heatmap analysis of γδ T cells expressing Th1 (IFNγ, IL-2, TNFα) and Th17 (IL-17A, IL-17F, IL-22) cytokines as well as cytotoxic (PFN, GZE B, GNLSN) and immune (GMCSF, PD-1, CD69) markers in LTB DM, LTB PDM, and LTB NDM comorbidities (**Figures 6A** **–C**). Our data show that the expression of cytokines and cytotoxic and immune markers were significantly different between the LTB (DM, PDM, NDM) comorbidities.

## Discussion

γδ T cells mainly contribute to anti-mycobacterial response built by other immune cells (Li and Wu, 2008; Brandes et al., 2009; Price and Hope, 2009). The activity of γδ T cells against Mtb occurred mostly *via* secreted cytokines, effector cytotoxic molecules, and chemotaxis. They were able to generate various cytokines that function against TB disease especially Th1, Th2, Th17 and regulatory cytokines (García et al., 1997; Tsukaguchi et al., 1999; Wesch et al., 2001). The role of γδ T cells expressing cytokine, cytotoxic, and other immune markers in LTB (NDM, PDM, and DM) comorbidities has not been studied and therefore was examined in the present study. Our data illustrate that γδ T cells expressing Th1 and Th17 cytokines as well as cytotoxic and immune markers were significantly decreased in LTB DM and/or LTB PDM compared to LTB NDM individuals.

γδ T cells can produce Th1 (TNFα, IFNγ) cytokines which are crucial for immune protection against viruses and intracellular microbes (O’Brien et al., 2009; Bonneville et al., 2010). Our previous studies have shown that antigen-specific Th1 responses in CD4^+^ T cells were reduced in LTB DM and LTB PDM comorbidities (Kumar et al., 2017). Our findings suggest that the frequencies of γδ T cells expressing Th1 cytokines (mainly IFNγ, TNFα) are also decreased in the LTB DM and LTB PDM groups compared to the LTB NDM group. The decreased frequencies might contribute to compromised immune protection in LTB individuals. Upon Mtb infection, γδ T cells activate Th17 cytokines and could proficiently control the effector role of other immune cells (Song et al., 2016). Specifically, IL-17-expressing γδ T cells could be involved in Mtb-mediated immune response associated with disease pathology in PTB disease (Umemura et al., 2007; van de Veerdonk et al., 2010). Similarly, IL-22 is produced by a wide range of cells, which includes Th1, Th17, γδ T cells, and innate immune cells like innate lymphoid cells (ILCs) (Toussirot et al., 2018; Cella et al., 2019). Our earlier study has shown that antigen-specific Th17 responses in CD4^+^ T cells were reduced in LTB DM and LTB PDM comorbidities (Kumar et al., 2017). Our current study demonstrates diminished frequencies of γδ T cells expressing IL-17 family cytokines in LTB DM or LTB PDM comorbidity. Thus, diminished protective cytokine production by γδ T cells could also contribute to diminished immune protection in these comorbidities.

γδ T cells confer to the primary immunity against Mtb disease by the production of cytokines and chemokines as well as stimulating cytotoxic molecules (Dieli et al., 2003; Qin et al., 2009). The important feature of cytotoxic markers (perforin, granzyme B, granulysin) is to lyse the Mtb-affected cells directly (Stenger, 2001). Our results exhibit that γδ T cell frequencies expressing perforin, granzyme B, granulysin, and perforin were significantly reduced upon PPD and WCL antigen stimulation in LTB (DM and PDM) coinfected individuals compared to LTB NDM individuals. This is in line with our previous study showing that CD8^+^ T cells expressing cytotoxic (perforin, granzyme B, CD107a) marker frequencies were diminished significantly in the LTB DM group when compared to the LTB NDM group (Kumar et al., 2015). In addition to the above cytokines and cytotoxic markers, we also studied immune (GMCSF, PD-1, CD69) markers and both PD-1 and CD69 were associated with significantly reduced frequencies in LTB DM and/or PDM compared to LTB NDM individuals. Thus, activation and cytotoxic marker expression are diminished in LTB DM and LTB PDM comorbidity.

We also show that the PBMC-stimulated supernatants of IFNγ, TNFα, and IL-17A cytokines were significantly increased in the DM and/or PDM comorbidities, indicating that the comorbidities might influence the disease pathogenesis in LTB individuals. Our study has certain limitations by showing only the baseline frequencies of LTB-coinfected individuals and not any follow-up frequencies of cytokines and cytotoxic markers upon treatment for DM. The other limitation is the sample size used in the study is small. Also, we did not examine the frequencies of γδ T cells expressing Th2 cytokines in LTB DM comorbidity in this study. Overall, our data reveal that DM and PDM comorbidities were characterized by decreased Th1, Th17, cytotoxic, and other immune markers and possibly associated with reduced immune protection in LTB infection.

## Data Availability Statement

The original contributions presented in the study are included in the article/**Supplementary Material**. Further inquiries can be directed to the corresponding author.

## Ethics Statement

The studies involving human participants were reviewed and approved by the National Institute of Research in Tuberculosis (NIRT) Internal Ethics Committee. The patients/participants provided their written informed consent to participate in this study.

## Author Contributions

Conceived and designed the experiments: GK and SB. Performed the experiments: GK, NP, and KM. Analyzed the data: GK. Contributed materials/reagents/analysis tools: PM and SB. Wrote the paper: GK and SB. All authors contributed to the article and approved the submitted version.

## Funding

The author (GK) thank the financial support from the DBT-RA Program in Biotechnology and Life Sciences. The funding was supported by the Division of Intramural Research, National Institute of Allergy and Infectious Diseases (NIAID), NIH. The funders have no role in the study design, data collection or analysis, data interpretation, or manuscript writing.

## Conflict of Interest

The authors declare that the research was conducted in the absence of any commercial or financial relationships that could be construed as a potential conflict of interest.

## Publisher’s Note

All claims expressed in this article are solely those of the authors and do not necessarily represent those of their affiliated organizations, or those of the publisher, the editors and the reviewers. Any product that may be evaluated in this article, or claim that may be made by its manufacturer, is not guaranteed or endorsed by the publisher.

## Supplementary Material

Supplementary Figure 1 Gating strategy of γδ T cells expressing Th1, Th17 cytokines, cytotoxic and immune markers. Single cells were gated from lymphocytes and further gated on CD3^+^ T cell population and γδ T cells were gated based on positive γδ TCR expression and the frequencies of different cytokines, cytotoxic and immune markers were shown.

Supplementary Figure 2 Representative plots for γδ T cells expressing **(A)** Th1 cytokines and GMCSF, **(B)** Th17 cytokines and PD-1, **(C)** cytotoxic markers and CD69 upon UNS, Mtb (PPD, WCL) antigen stimulation and positive antigen (P/I) control stimulation.
