# Supplementary figures and images for: Decreased Frequencies of Gamma/Delta T Cells Expressing Th1/Th17 Cytokine, Cytotoxic, and Immune Markers in Latent Tuberculosis-Diabetes/Pre-Diabetes Comorbidity
Source: Front Cell Infect Microbiol. 2021 Oct 26;11:756854. doi: 10.3389/fcimb.2021.756854 (PMC8577793; doi:10.3389/fcimb.2021.756854)

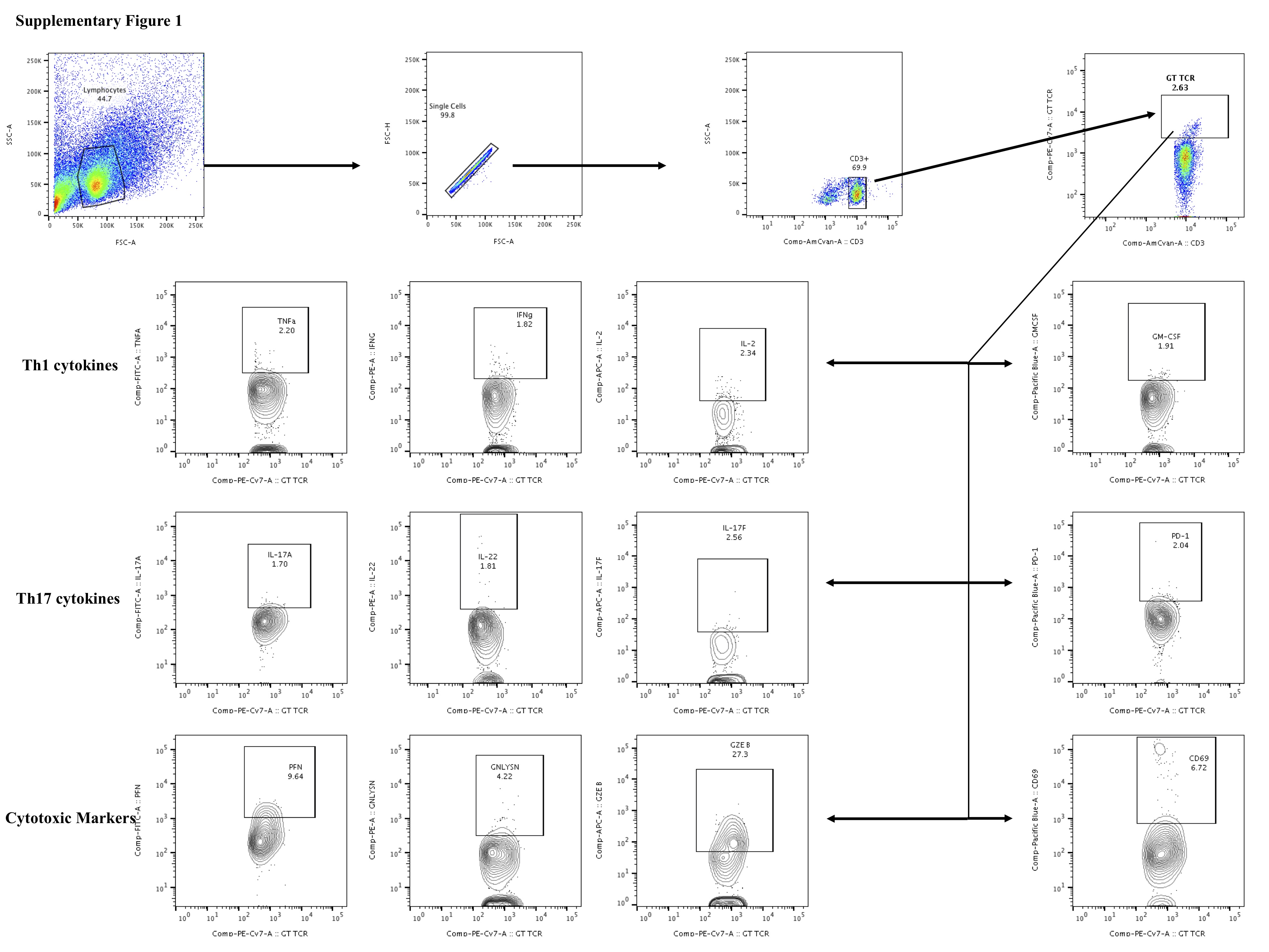

Supplement: Supplementary Figure 1 — Gating strategy of γδ T cells expressing Th1, Th17 cytokines, cytotoxic and immune markers. Single cells were gated from lymphocytes and further gated on CD3+ T cell population and γδ T cells were gated based on positive γδ TCR expression and the frequencies of different cytokines, cytotoxic and immune markers were shown. [file Image_1.jpeg]

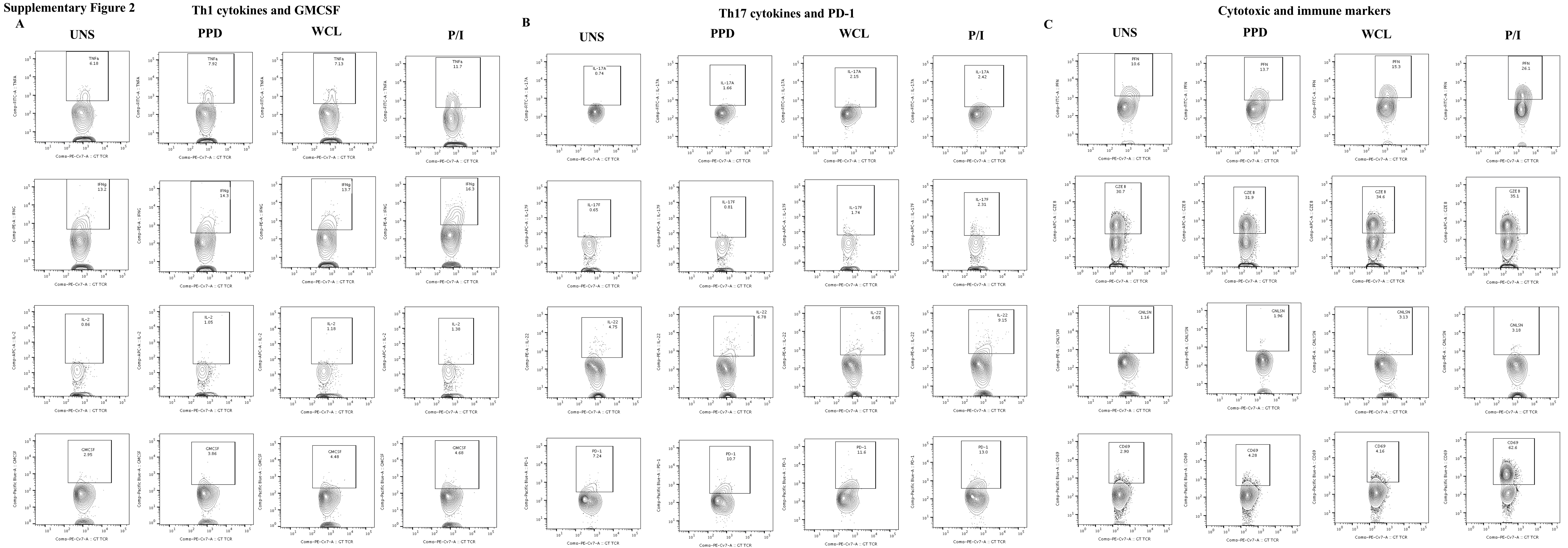

Supplement: Supplementary Figure 2 — Representative plots for γδ T cells expressing (A) Th1 cytokines and GMCSF, (B) Th17 cytokines and PD-1, (C) cytotoxic markers and CD69 upon UNS, Mtb (PPD, WCL) antigen stimulation and positive antigen (P/I) control stimulation. [file Image_2.jpeg]
